# Sleep Architecture, Obstructive Sleep Apnea, and Cognitive Function in Adults

**DOI:** 10.1001/jamanetworkopen.2023.25152

**Published:** 2023-07-18

**Authors:** Matthew P. Pase, Stephanie Harrison, Jeffrey R. Misialek, Christopher E. Kline, Marina Cavuoto, Andree-Ann Baril, Stephanie Yiallourou, Alycia Bisson, Dibya Himali, Yue Leng, Qiong Yang, Sudha Seshadri, Alexa Beiser, Rebecca F. Gottesman, Susan Redline, Oscar Lopez, Pamela L. Lutsey, Kristine Yaffe, Katie L. Stone, Shaun M. Purcell, Jayandra J. Himali

**Affiliations:** 1 Turner Institute for Brain and Mental Health, Monash University, Melbourne, Victoria, Australia; 2 Harvard T.H. Chan School of Public Health, Massachusetts; 3 Framingham Heart Study, Framingham, Massachusetts; 4 California Pacific Medical Center, Research Institute, San Francisco; 5 Division of Epidemiology and Community Health, University of Minnesota, Minneapolis; 6 Department of Health and Human Development, University of Pittsburgh, Pittsburgh, Pennsylvania; 7 Douglas Mental Health University Institute, McGill University, Montreal, Quebec, Canada; 8 Division of Sleep and Circadian Disorders, Brigham and Women’s Hospital, Boston, Massachusetts; 9 Department of Medicine, Harvard Medical School, Boston, Massachusetts; 10 Department of Psychiatry and Behavioral Sciences, University of California; 11 Department of Neurology, Boston University School of Medicine, Boston, Massachusetts; 12 Department of Biostatistics, Boston University School of Public Health, Boston, Massachusetts; 13 Glenn Biggs Institute for Alzheimer’s and Neurodegenerative Diseases, University of Texas Health Science Center San Antonio; 14 National Institute of Neurological Disorders and Stroke Intramural Research Program, Bethesda, Maryland; 15 Department of Neurology, University of Pittsburgh, Pittsburgh, Pennsylvania; 16 Department of Psychiatry, University of Pittsburgh, Pittsburgh, Pennsylvania; 17 Department of Psychiatry, University of California, San Francisco; 18 Department of Neurology, University of California, San Francisco; 19 Department of Epidemiology, University of California, San Francisco; 20 Department of Epidemiology and Biostatistics, University of California, San Francisco; 21 Department of Psychiatry, Brigham and Women’s Hospital, Boston, Massachusetts; 22 Department of Population Health Sciences, University of Texas Health Science Center, San Antonio

## Abstract

**Importance:**

Good sleep is essential for health, yet associations between sleep and dementia risk remain incompletely understood. The Sleep and Dementia Consortium was established to study associations between polysomnography (PSG)–derived sleep and the risk of dementia and related cognitive and brain magnetic resonance imaging endophenotypes.

**Objective:**

To investigate association of sleep architecture and obstructive sleep apnea (OSA) with cognitive function in the Sleep and Dementia Consortium.

**Design, Setting, and Participants:**

The Sleep and Dementia Consortium curated data from 5 population-based cohorts across the US with methodologically consistent, overnight, home-based type II PSG and neuropsychological assessments over 5 years of follow-up: the Atherosclerosis Risk in Communities study, Cardiovascular Health Study, Framingham Heart Study (FHS), Osteoporotic Fractures in Men Study, and Study of Osteoporotic Fractures. Sleep metrics were harmonized centrally and then distributed to participating cohorts for cohort-specific analysis using linear regression; study-level estimates were pooled in random effects meta-analyses. Results were adjusted for demographic variables, the time between PSG and neuropsychological assessment (0-5 years), body mass index, antidepressant use, and sedative use. There were 5946 participants included in the pooled analyses without stroke or dementia. Data were analyzed from March 2020 to June 2023.

**Exposures:**

Measures of sleep architecture and OSA derived from in-home PSG.

**Main Outcomes and Measures:**

The main outcomes were global cognitive composite *z* scores derived from principal component analysis, with cognitive domains investigated as secondary outcomes. Higher scores indicated better performance.

**Results:**

Across cohorts, 5946 adults (1875 females [31.5%]; mean age range, 58-89 years) were included. The median (IQR) wake after sleep onset time ranged from 44 (27-73) to 101 (66-147) minutes, and the prevalence of moderate to severe OSA ranged from 16.9% to 28.9%. Across cohorts, higher sleep maintenance efficiency (pooled β per 1% increase, 0.08; 95% CI, 0.03 to 0.14; *P* < .01) and lower wake after sleep onset (pooled β per 1-min increase, −0.07; 95% CI, −0.13 to −0.01 per 1-min increase; *P* = .02) were associated with better global cognition. Mild to severe OSA (apnea-hypopnea index [AHI] ≥5) was associated with poorer global cognition (pooled β, −0.06; 95% CI, −0.11 to −0.01; *P* = .01) vs AHI less than 5; comparable results were found for moderate to severe OSA (pooled β, −0.06; 95% CI, −0.11 to −0.01; *P* = .02) vs AHI less than 5. Differences in sleep stages were not associated with cognition.

**Conclusions and Relevance:**

This study found that better sleep consolidation and the absence of OSA were associated with better global cognition over 5 years of follow-up. These findings suggest that the role of interventions to improve sleep for maintaining cognitive function requires investigation.

## Key Points

**Question:**

Which aspects of sleep architecture and respiratory-related sleep disturbances are associated with cognitive function in middle-aged to older adults?

**Findings:**

This study of 5946 adults in 5 independent community-based cohorts with an overnight sleep study and neuropsychological assessments found that better sleep consolidation and the absence of sleep apnea were associated with better global cognition, whereas individual sleep stage percentages were not.

**Meaning:**

These findings suggest that in adults without dementia, sleep consolidation and the absence of sleep apnea may be particularly important for optimizing cognition with aging.

## Teaser

This cohort study investigates the association of sleep architecture and obstructive sleep apnea measures with cognitive function among middle-aged to older adults in 5 cohorts.

## Introduction

Sleep of sufficient quality and duration may be associated with decreased risk of dementia through several mechanisms, including augmenting the glymphatic clearance of Alzheimer disease proteins,^1,2^ facilitating memory consolidation and synaptic remodeling,^3,4,5,6^ and reduced risk of cardiometabolic diseases and vascular brain injury,^7,8^ which are known factors associated with dementia risk.^9,10,11^ However, the association between sleep and cognitive impairment remains equivocal; poor sleep was not among 12 modifiable risk factors outlined in the Lancet Commission on dementia prevention.^11^

Much of the uncertainty surrounding associations between sleep and dementia arises from a paucity of data with long follow-up durations, objective measures of sleep, or consistent data formats and analysis methods to facilitate pooling and sharing of data across studies.^12^ To address this gap, we created the Sleep and Dementia Consortium to advance sleep research to inform new strategies for dementia prevention.

This article aimed to investigate associations of sleep macroarchitecture and obstructive sleep apnea (OSA) with cognition over 5 years of follow-up across participating cohorts of the Sleep and Dementia Consortium. By combining across studies, this analysis attempted to address several unanswered questions, including which sleep variables are most robustly associated with cognition, what cognitive domains are most sensitive to differences in sleep, and whether there are systematic differences by key variable, such as sex and *APOE* genotype. Based on prior work, it was hypothesized that a greater percentage of time in rapid eye movement (REM) sleep and higher sleep consolidation would be associated with better cognition.^13,14,15,16^

## Methods

All participants in this cohort study provided written informed consent before study commencement. Each cohort obtained institutional review board (IRB) approval at its respective institution, and this study was approved by the University of Texas Health Science Center at San Antonio IRB. This report follows the Strengthening the Reporting of Observational Studies in Epidemiology (STROBE) reporting guideline for cohort studies.

### Study Design

The Sleep and Dementia Consortium comprises 5 prospective community-based cohorts that have performed methodologically consistent, overnight, home-based polysomnography (PSG) and neurocognitive assessments. Given that this is the first study from the Sleep and Dementia Consortium, a broad overview, including its design, goals, participating cohorts, and methods is shown in the eMethods in Supplement 1. For this analysis, we investigated associations between baseline sleep metrics and cognitive function measured within the subsequent 5 years. Sleep measures from the baseline PSG were used from each cohort. The timing of assessments is shown in the eMethods in Supplement 1. Cohorts include the Atherosclerosis Risk in Communities (ARIC) study, Cardiovascular Health Study (CHS), Framingham Heart Study (FHS), Osteoporotic Fractures in Men Study (MrOS), and Study of Osteoporotic Fractures (SOF).

### Participants

Enrollment and cohort design methods have been published previously (ARIC,^17,18,19^ CHS,^20,21,22^ FHS,^23,24^ MrOS,^25,26^ and SOF^27,28,29^) and are summarized in the eMethods in Supplement 1. This analysis was limited to participants aged at least 45 years who were free of dementia and stroke and who had baseline PSG and cognitive testing within 5 years after PSG. To reduce the influence of potentially spurious data, participants with less than 180 minutes of total sleep time or less than 1 minute of REM sleep were excluded. Sample selection across cohorts is shown in eTable 1 in Supplement 1.

### Sleep Assessments

Participants in the ARIC, CHS, and FHS completed in-home PSG as part of the Sleep Heart Health Study, a consortium of prospective cohort studies established to examine sleep disorders as risk factors associated with cardiovascular disease and stroke.^30^ All cohorts used a standardized protocol to complete overnight, home-based type II PSGs between 1995 and 1998. Participants from each cohort were invited to complete an initial home-based PSG using the Compumedics P Series System (Abbotsford). Electroencephalogram (C3A2 and C4A1), electrooculogram, electromyogram, thoracic and abdominal displacement (inductive plethysmography bands), airflow (nasal-oral thermocouples), finger pulse oximeter, a single bipolar electrocardiogram, body position by a mercury gauge sensor, and ambient light level were all recorded.

The same central Sleep Reading Center oversaw training, conduct and analysis of all sleep studies. Methods, including scoring guidelines and reliability, have been previously published.^30,31,32^ The ARIC, CHS, and FHS sleep studies were standardized as part of the Sleep Heart Health Study. For SOF and MrOS, sleep protocols were modeled on the Sleep Heart Health Study, enabling effective harmonization and cross-study comparison. SOF and MrOS added nasal cannula pressure and bilateral piezoelectric sensors to detect leg movements. Sleep was scored in 30-second epochs by trained PSG technicians blinded to other data according to established guidelines (Rechtschaffen and Kales^33^ and American Sleep Disorders Association arousal criteria^34^), with excellent interscorer and intrascorer reliability on epoch-by-epoch sleep staging (κ statistics >0.80) and respiratory-related measures.^31,32^ The following variables were calculated for each participant.

### Measures of Sleep Macroarchitecture and Daytime Sleepiness

Stage 1 (N1%), stage 2 (N2%), stage 3 (N3%), and REM (REM%) sleep were defined as the duration of each sleep stage expressed as a percentage of total sleep time. Wake after sleep onset was defined as the number of minutes spent awake between sleep onset and offset. Total sleep time was defined as the number of hours asleep between sleep onset and offset. Sleep maintenance efficiency was expressed as the ratio of total sleep time to the sleep period (defined as the time between sleep onset and offset), given as a percentage. The Epworth Sleepiness Scale was used to measure daytime sleepiness. The ESS is an 8-item questionnaire that measures daytime sleepiness. Each item is scored on a 4-point scale (response range, 0-3) to examine the likelihood of falling asleep during 8 different situations. Higher scores indicate higher daytime sleepiness.

### Measures of Obstructive Sleep Apnea

The apnea-hypopnea index (AHI) was defined as the number of obstructive sleep apneas (OSAs) plus the number of hypopneas accompanied by a greater than 30% reduction in airflow and 4% or greater oxygen desaturation or arousal per hour of sleep. Sleep time with arterial oxygen saturation less than 90% was defined as the ratio of the number of minutes with arterial oxygen saturation less than 90% to the total sleep time expressed in hours. Sleep variables were calculated centrally to ensure consistency of analysis and effective harmonization.

### Cognitive Assessments

The primary outcome was global cognition. Cognitive domain scores were investigated as secondary outcomes. Units were expressed as *z* scores, with higher values indicating better performance.

Methods to assess cognition varied across cohorts. Analyses of hundreds of independent data sets have shown that a single cognitive factor can explain more than 40% of variance across cognitive test batteries.^35^ Moreover, performance on any given cognitive test partly depends on a general cognitive ability.^36^ Given that all cognitive tests load onto a latent general cognitive ability, one common approach to estimate general cognitive function is to perform a principal component analysis of cognitive test scores, extracting the first unrotated component. Thus, even though cognitive tests differ across cohorts, this approach allows for estimating general cognitive function across studies. To this end, the investigators have already created a general cognitive score for ARIC, CHS, and FHS as part of a previous meta-analysis.^36^

The method involved calculating a general cognitive phenotype from at least 3 different cognitive tests in each cohort. Multiple outcomes from the same cognitive test were not included in the computation. Principal component analysis was applied to the cognitive test scores to derive a general cognitive score by forcing a single-factor solution. The same methods were applied to MrOS and SOF to generate global cognition across all cohorts. eTable 2 in Supplement 1 provides further details, including individual tasks used to create the global cognitive composite for each cohort and their factor loadings. In a secondary analysis, we used a neuropsychological framework to group cognitive tasks into broad cognitive domains of executive function, attention and processing speed, verbal learning and memory, language, and visuospatial function. Individual cognitive test outcomes were converted to *z* scores based on the cohort-specific sample mean and SD and the mean found within each domain. The organization of cognitive tests into broad cognitive domains was completed by iterative consensus among neuropsychologists using a shared neuropsychological framework of cognitive domains (eTable 3 in Supplement 1). Tasks for which higher scores indicate poorer performance were reverse-coded such that higher scores always indicated better performance.

### Covariates

The following covariates were selected based on expert knowledge and prior literature of known confounders of sleep and cognition: age (years), age-squared, sex (male and female), education (<high school [<12 years], high school [12 years], and >high school [>12 years]), the time between PSG and neuropsychological assessment (years), body mass index (BMI; calculated as weight in kilograms divided by height in meters squared), antidepressant use (yes vs no), and sedative use (yes vs no). Covariates were obtained at the time of PSG or the clinic exam closest to the PSG and were included in all statistical models. To characterize cohorts, data were obtained on clinical characteristics and demographics, including self-reported race and ethnicity. Sources of race and ethnicity classifications and available categories varied by study (eMethods in Supplement 1). Analyses in this study were limited to Black, White, and other race or ethnicity due to data availability. Race and ethnicity assessments were included in this analysis to determine how representative cohort populations were of the broader US population.

### Statistical Analysis

Table 1 summarizes sleep measures used in analyses. Statistical analysis was performed using SAS statistical software version 9.4 (SAS Institute) and R statistical software version 4.3.0 (R Project for Statistical Computing). Associations between each sleep variable and each cognitive outcome were investigated in separate linear regression, adjusting for covariates. Cohort-specific analyses were conducted for each cohort with study-level estimates pooled centrally in random effects meta-analyses using the Der Simonian and Laird inverse-variance method. The Higgins *I*^2^ test was used to test for heterogeneity in effect sizes.^37^ We did not conduct a meta-analysis of results for the cognitive domain of language given that only 2 cohorts (CHS and FHS) contributed data. Statistical tests were 2-sided, and results were considered significant if *P* < .05. Missing data, of which there was little (eTable 1 in Supplement 1), were excluded from the analysis. Data were analyzed from March 2020 to June 2023.

**Table 1.** Sleep Characteristics Across Cohorts

Sleep exposure	Data manipulation^a^	Participants, No (%) (N = 5946)^b^				
		ARIC (n = 1791)	CHS (n = 701)	FHS (n = 640)	MrOS (n = 2619)	SOF (n = 195)
Continuous variables						
N1, median (IQR), %	Square root	4.6 (2.9-7.0)	4.4 (2.7-6.5)	4.5 (2.6-7.2)	5.8 (4.0-8.5)	4.4 (2.9-6.0)
N2, mean (SD), %	NA	55.6 (11.3)	57.4 (12.6)	55.5 (11.1)	62.6 (9.5)	55.8 (11.8)
N3, median (IQR), %	Square root	17.9 (9.5-25.7)	16.5 (7.8-26.1)	18.2 (11.0-25.8)	11.0 (3.8-16.7)	18.1(11.4-25.9)
REM, mean (SD), %	NA	20.7 (5.8)	19.2 (6.3)	20.7 (5.7)	19.5 (6.4)	19.8 (7.0)
Sleep maintenance efficiency, median (IQR), %	NA	88.3 (82.0-92.6)	85.4 (77.6-91.2)	89.9 (83.7-93.6)	78.5 (70.3-85.1)	82.6 (73.0-88.3)
Wake after sleep onset, median (IQR), min	Natural log	49.5 (30.5-77.5)	62.0 (36.0-97.0)	44.0 (27.3-73.0)	101.0 (66.0-147.0)	77.0 (52.0-116.0)
Categorical variables						
Mild to severe OSA	Dichotomized, apnea hypopnea index <5 (reference) vs ≥5	800 (49.6)	376 (61.2)	270 (45.2)	1674 (63.9)	110 (56.4)
Moderate to severe OSA	Dichotomized, apnea hypopnea index <15 (reference) vs ≥15	317 (19.6)	159 (25.9)	101 (16.9)	756 (28.9)	39 (20.0)
Sleep time with arterial oxygen saturation <90%	Dichotomized, <1% (reference) vs ≥1%	530 (29.6)	274 (39.2)	181 (28.3)	1351 (51.6)	80 (41.03)
Total sleep time	Dichotomized, ≤6 h (reference) vs >6-9 h^c^	1054 (58.9)	353 (50.4)	424 (66.2)	1334 (50.9)	102 (52.3)

Abbreviations: ARIC, Atherosclerosis Risk in Communities study; CHS, Cardiovascular Health Study; FHS, Framingham Heart Study; MrOS, Osteoporotic Fractures in Men Study; N1, stage 1 sleep; N2, stage 2 sleep; N3, stage 3 sleep; NA, not applicable; OSA, obstructive sleep apnea; REM, rapid eye movement; SOF, Study of Osteoporotic Fractures.

^a^ For data manipulation used in analysis, original units are presented for continuous measures.

^b^ Denominators varied by specific variable for each study.

^c^ The first sleep recording in the Sleep Heart Health Study was limited by battery life to 9 hours, meaning sleep durations greater than 9 hours could not be examined across cohorts.

We explored effect modification by sex (male vs female), *APOE ε4* allele status (carrier vs noncarrier), and excessive daytime sleepiness (Epworth Sleepiness Scale scores ≥11 vs <11) for the primary outcome of global cognition. In the presence of an interaction (*P* < .05), results were stratified at each level of the moderating variable. Interaction results were not pooled in a meta-analysis. Instead, we interpreted patterns that were evident across studies. Sex was not examined as a moderating variable in MrOS or SOF given that these cohorts were exclusively male and female, respectively, and *APOE* genotype was not examined as a moderating variable in SOF given that it was not available for all participants.

## Results

### Participant Characteristics

Across cohorts, there were 5946 participants (1875 females [31.5%]; mean age range, 58-89 years at the time of PSG; 232 Black [3.9%]; 5459 White [91.7%], and 255 other race or ethnicity [4.3%]), including 1791 participants in ARIC, 701 participants in CHS, 640 participants in FHS, 2619 participants in MrOS, and 195 participants in SOF. Most participants were male given that the large MrOS study was only in males (Table 2). Overall, 495 participants (8.3%) did not have a high school degree and 1298 participants (21.8%) reported using sleeping pills regularly.

**Table 2.** Cohort Characteristics

Covariate	Participants, No. (%) (N = 5946)				
	ARIC (n = 1791)	CHS (n = 701)^a^	FHS (n = 640)	MrOS (n = 2619)	SOF (n = 195)
Age, mean (SD), y	62.4 (5.7)	76.7 (3.7)	58.5 (8.4)	76.2 (5.5)	89.1 (2.9)
Sex					
Female	927 (51.8)	408 (58.2)	345 (53.9)	0 (0)	195 (100)
Male	864 (48.2)	293 (41.8)	295 (46.1)	2619 (100)	0 (0)
Self-reported race and ethnicity, No. (%)^b^					
Black	7 (0.4)	100 (14.3)	54 (8.4)	81 (3.1)	15 (7.7)
White	1778 (99.3)	601 (85.7)	518 (80.9)	2382 (91.0)	179 (91.8)
Other	6 (0.3)	0 (0)	68 (10.6)	156 (5.9)	1 (0.5)
American Indian or Alaska Native	1 (<0.1)	NA	0 (0)	1 (0)	NA
Asian	5 (0.3)	NA	0 (0)	76 (2.9)	1 (0.5)
Asian Indian or Pacific Islander	NA	NA	22 (3.4)	NA	NA
Hispanic	NA	NA	46 (7.2)	48 (1.8)	NA
Native Hawaiian or Pacific Islander	NA	NA	0 (0)	3 (0.1)	NA
Multiracial or multiethnic	NA	NA	0 (0)	28(1.1)	NA
Education					
<High school	185 (10.3)	118 (16.8)	47 (7.4)	114 (4.4)	31 (17.2)
High school	641 (35.8)	394 (56.2)	173 (27.0)	420 (16.0)	89 (49.4)
>High school	965 (53.9)	189 (27.0)	420 (65.6)	2085 (79.6)	60 (33.3)
Time between PSG and cognitive testing, mean (SD), y	0.3 (0.8)	2.1 (0.6)	3.7 (1.4)	0 (0)	4.63 (0.7)
Systolic BP, mean (SD), mm Hg	121.0 (17.0)	129.6 (18.8)	125.9 (16.7)	126.7 (16.1)	135.8 (16.7)
Hypertension treatment	606 (33.8)	381 (54.4)	158 (24.7)	1735 (66.3)	140 (71.8)
Hypertension	632 (35.3)	392 (55.9)	242 (37.9)	1876 (71.6)	159 (82.0)
Prevalent diabetes	98 (5.5)	78 (11.1)	62 (9.8)	342 (13.1)	18 (9.2)
Prevalent CVD	143 (8.0)	69 (9.8)	42 (6.6)	1032 (39.5)	36 (20.0)
Current smoker	176 (9.8)	38 (5.4)	85 (13.3)	1577 (60.2)	3 (1.5)
BMI, median (IQR)	28.3 (25.3-31.7)	27.4 (25.0-29.8)	27.3 (24.5-30.9)	26.7 (24.6-29.3)	27.8 (25.2-31.3)
Sleeping pill use	434 (24.2)	156 (22.3)	116 (18.4)	541 (20.7)	51 (26.2)
Antidepressant use	128 (7.2)	34 (4.9)	38 (5.9)	192 (7.3)	15 (7.7)
Sedative use	86 (4.8)	42 (6.0)	24 (3.8)	183 (7.0)	28 (14.4)
*APOE ε4* carrier	483 (27.0)	162 (23.1)	137 (21.9)	497 (23.6)	NA

Abbreviations: ARIC, Atherosclerosis Risk in Communities study; BMI, body mass index (calculated as weight in kilograms divided by height in meters squared); BP, blood pressure; CHS, Cardiovascular Health Study; CVD, cardiovascular disease; FHS, Framingham Heart Study; MrOS, Osteoporotic Fractures in Men Study; NA, not applicable; SOF, Study of Osteoporotic Fractures.

^a^ Based on the sample of 701 participants with data available for global cognition. Analysis of domain-specific scores was based on a subset of this sample with more extensive cognitive testing (232 participants).

^b^ Race and ethnicity were self-reported and categorized as Black, White, or other, with other defined by different cohorts as American Indian or Alaska Native, Asian, Asian Indian or Pacific Islander, Hispanic, Native Hawaiian or Pacific Islander, or multiracial.

The percentage of people with at least mild OSA (AHI ≥5) ranged from 45.2% in the FHS to 63.9% in MrOS (Table 1), reflecting differences in age and sex of these cohorts. Similarly, the percentage of people with at least moderate OSA (AHI ≥15) was lowest in the FHS (16.9%) and highest in MrOS (28.9%). Median sleep efficiency was highest among participants in the FHS and lowest among participants in MrOS (Table 1). Raw cognitive test means for each cohort are shown in eTable 4 in Supplement 1.

### Associations Between Sleep Metrics and Global Cognition

Across cohorts, higher sleep maintenance efficiency (pooled β per 1% increase, 0.08; 95% CI, 0.03 to 0.14; *P* < .01) and lower wake after sleep onset (pooled β per 1-min increase, −0.07 ; 95% CI, −0.13 to −0.01; *P* = .02) were independently associated with superior global cognition (Figure). Sleep stage percentages were not associated with global cognition across cohorts, while persons with mild to severe OSA (AHI ≥5) displayed poorer global cognitive function vs AHI <5 (pooled β, −0.06; 95% CI, −0.11 to −0.01; *P* = .01) (Figure). A comparable outcome was observed for persons with at least moderate OSA (AHI ≥15) vs AHI less than 5 (pooled β, −0.06; 95% CI, −0.11 to −0.01; *P* = .02). Sleep time with oxygen saturation less than 90% was not associated with global cognitive function.

**Figure.**
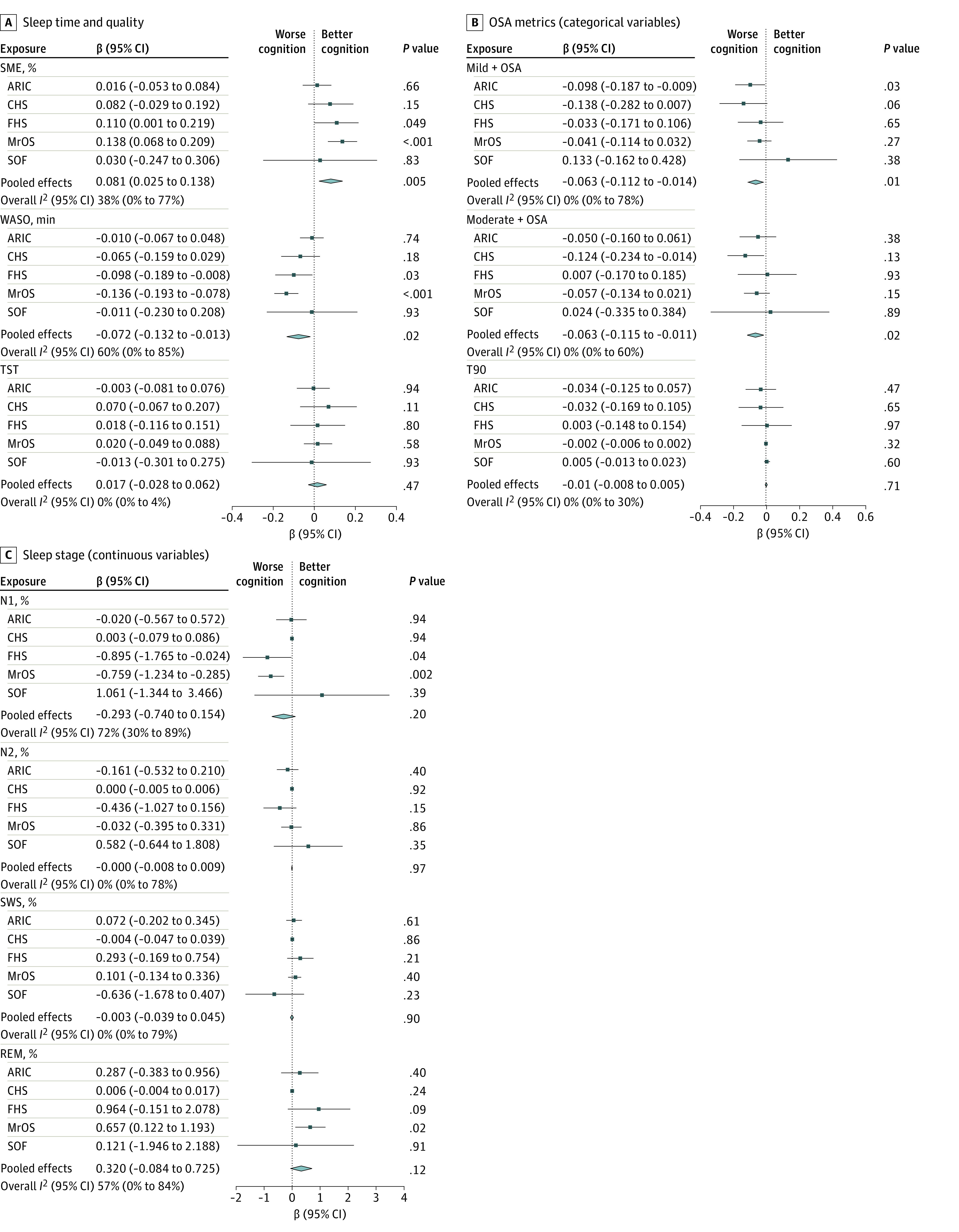
Pooled Associations Between Each Sleep Measure and Global Cognition ARIC indicates Atherosclerosis Risk in Communities study; CHS, Cardiovascular Health Study; FHS, Framingham Heart Study; MrOS, Osteoporotic Fractures in Men Study; N1, stage N1 sleep; N2, stage N2 sleep; OSA, obstructive sleep apnea; REM, rapid eye movement; SME, sleep maintenance efficiency; SOF, Study of Osteoporotic Fractures; SWS, slow wave sleep; T90, percentage of sleep time with oxygen saturation less than 90%; TST, total sleep time; WASO, wake after sleep onset.

### Associations Between Sleep Metrics and Cognitive Domains

Pooled estimates revealed that short total sleep time was associated with poorer attention and processing speed (pooled β, 0.07; 95% CI, 0.02-0.11 for total sleep time of >6 hours vs ≤6 hours; *P* < .01) (eTable 5 in Supplement 1). Pooled effects did not show overall associations between sleep and learning and memory or visuospatial abilities (eTables 6-9 in Supplement 1).

### Exploration of Interaction Associations

In the CHS, the association between REM sleep percentage and global cognition differed by sex, with a positive association observed in females (β per 1% increase, 0.02; 95% CI, −1.25 to 1.48; *P* = .01) and no association observed in men (β per 1% increase, −0.01; 95% CI, −1.66 to 1.54; *P* = .25; *P* for interaction = .01) (eTable 11 in Supplement 1). In the FHS, the association between moderate to severe OSA (AHI ≥15 vs <5) and global cognition differed by excessive daytime sleepiness; OSA was associated with poorer global cognition in persons with (β, −0.54; 95% CI, −0.96 to −0.11; *P* = .01) but not those without (β, 0.16; 95% CI, −0.05 to 0.37; *P* = .14) excessive daytime sleepiness (*P* for interaction = .006) (eTable 12 in Supplement 1). All other sleep-cognition associations were similar across interaction variables (eTables 10-12 in Supplement 1).

## Discussion

In this cohort study, we examined associations between sleep and cognition in the Sleep and Dementia Consortium. Results demonstrated that poorer sleep consolidation and prevalent OSA were associated with poorer global cognition within 5 years. Sleep stage percentages were not associated with global cognition across cohorts. We found 1 association between sleep metrics and individual cognitive domains: normal sleep duration compared with short sleep duration was associated with better attention and processing speed.

Previous research has found an association between the presence of OSA and poorer performance across several cognitive domains^38,39^; however, OSA was associated only with global cognition in this study. This study’s finding of an association between mild OSA and poorer cognition in persons who did not present to a sleep clinic is an important observation. Persons with OSA diagnosed based on incidental findings compared with patients referred clinically for PSG may differ across several characteristics, including comorbidities, overall dementia risk factor burden, and the severity of sleep disturbances. Although there are direct (eg, intermittent hypoxia leading to ischemic brain injury or sleep fragmentation) and indirect (eg, systemic inflammation or cardiovascular instability) mechanisms that may link OSA with poorer cognition, no conclusions regarding causation can be made from this observational study.

Findings of associations of sleep quality, sleep consolidation, and normal sleep time (compared with short sleep time) with better cognition are consistent with similar work that examined sleep using self-report and actigraphy.^13,40,41,42,43,44^ The lack of an association between sleep architecture and verbal learning and memory in our study was interesting given the role of sleep in memory consolidation.^4^ However, it should be noted that we did not examine sleep-dependent learning specifically (ie, the presentation of stimuli and the recall of that stimuli were not separated by an overnight sleep period). Moreover, the role of sleep in the consolidation of episodic memory may be greatest in younger adults,^45^ and this sample comprised middle-aged to older adults.

Previously, we showed that a lower level of REM sleep was associated with cognitive decline in MrOS^14^ and a higher risk of incident dementia in the FHS.^15^ In this study, a higher REM percentage was associated with better cognition in MrOS; in the FHS, there was no such association. However, the pooled estimate across cohorts did not show an association between REM percentage and cognition. Additional analysis in the Sleep and Dementia Consortium with other related outcomes (eg, risk of incident dementia) may help clarify REM’s role in cognitive aging and dementia.

Considerable interest surrounds the role of N3 sleep in dementia given that glymphatic clearance is optimized in N3 sleep^1^ and N3 sleep plays a pivotal role in memory consolidation.^4,46^ Based on these mechanisms, enhancing slow waves has been proposed as 1 potential therapy for mitigating cognitive decline.^47^ However, we did not find an association between differences in N3 percentage and cognitive function, even when examining individual study-level estimates. These results are consistent with those of several smaller studies in older adults^48^ and earlier findings from the FHS and MrOS whereby differences in N3 sleep time or percentage were not associated with cognition or the risk of incident dementia on follow-up.^14,15,16^ It is possible that other sleep stages (eg, REM)^49^ compensate for age-related declines in N3 or that single-night PSG is insufficient to quantify N3 sleep, which may also reflect increased homeostatic drive after relative sleep deprivation before the sleep study. Lastly, the association between N3 and cognition may differ by variables not considered here, such as cognitive status or brain amyloid burden.^50^ As shown previously, cognition may be associated with specific features of non-REM sleep, including slow oscillations and spindles, beyond non-REM macroarchitecture.^16^

Sleep and cognition are dynamic across the life span. However, it is unclear if there are sensitive periods in adult life during which good sleep is more critical for preventing late-life cognitive impairment or whether it is the duration of exposure to suboptimal sleep that is associated with cognitive outcomes. Cohorts that contributed to the Sleep and Dementia Consortium differed in population characteristics, including age and sex distributions. This allowed effect sizes to be directly compared and contrasted across cohorts with these different characteristics. For example, associations between OSA metrics and global cognition often trended against the expected direction of associations in the youngest (FHS) and oldest (SOF) cohorts. Many factors may underly differences in results between studies. However, like many established dementia risk factors,^51^ sleep and cognition associations may be dynamic across the life span. Interestingly, we did not find consistent patterns of interaction associations by sex, *APOE* ε4 status, or excessive daytime sleepiness. Thus, associations between sleep and cognition appear to be relatively consistent across different levels of these variables.

### Strengths and Limitations

Strengths of the current study include the large, pooled sample size, objective assessment of sleep in the participant’s home, and characterization of cognitive domains. A further strength was the central harmonization of sleep variables and covariates. However, this study is not without limitations. Sleep and cognition were assessed at 1 time. Furthermore, given that associations between sleep and brain health are likely bidirectional, longer follow-up durations may be required to tease apart temporal associations between poor sleep and the development of cognitive impairment. We plan to address these limitations in future Sleep and Dementia Consortium studies involving analyses of PSG at 2 times and incident dementia follow-up.

## Conclusions

In this study of participants from the Sleep and Dementia Consortium, evidence from multiple population-based cohorts indicated that better sleep consolidation and the absence of OSA were associated with superior general cognitive function. No associations were found between sleep stage percentages and cognition. Moreover, there was little evidence to suggest that sex, *APOE ε4*, or excessive daytime sleepiness interacted with associations. With respect to individual cognitive domains, only short sleep duration was associated with poorer attention and processing speed. Future Sleep and Dementia Consortium analyses will build upon these findings to further investigate whether and how poor sleep may be associated with cognitive impairment and dementia.

## Notes

Supplement 1. **eMethods.** The Sleep and Dementia Consortium (SDC): Design and Overarching Aims **eTable 1.** Analytic Sample Selection **eTable 2.** Creation of Global Cognitive Composite Scores **eTable 3.** Cognitive Domain Scores Used in Analyses **eTable 4.** Means and SDs for Each Cognitive Test in Each Cohort Analysis Sample **eTable 5.** Association Between Sleep and Attention and Processing Speed Across Cohorts **eTable 6.** Association Between Sleep and Executive Function Across Cohorts **eTable 7.** Association Between Sleep and Verbal Learning and Memory Across Cohorts **eTable 8.** Association Between Sleep and Language Across Cohorts **eTable 9.** Association Between Sleep and Visuospatial Function Across Cohorts **eTable 10.** Interactions by Sleep and APOE for Global Cognition **eTable 11.** Interactions by Sleep and Sex for Global Cognition **eTable 12.** Interaction by Sleep and Excessive Daytime Sleepiness and Global Cognition
eReferences.

Supplement 2.
Data Sharing Statement
