# Supplementary material for: Sleep Architecture, Obstructive Sleep Apnea, and Cognitive Function in Adults
Source: JAMA Netw Open. 2023 Jul 18;6(7):e2325152. doi: 10.1001/jamanetworkopen.2023.25152 (PMC10354680; doi:10.1001/jamanetworkopen.2023.25152)
Supplement: Supplement 1. — eMethods. The Sleep and Dementia Consortium (SDC): Design and Overarching Aims eTable 1. Analytic Sample Selection eTable 2. Creation of Global Cognitive Composite Scores eTable 3. Cognitive Domain Scores Used in Analyses eTable 4. Means and SDs for Each Cognitive Test in Each Cohort Analysis Sample eTable 5. Association Between Sleep and Attention and Processing Speed Across Cohorts eTable 6. Association Between Sleep and Executive Function Across Cohorts eTable 7. Association Between Sleep and Verbal Learning and Memory Across Cohorts eTable 8. Association Between Sleep and Language Across Cohorts eTable 9. Association Between Sleep and Visuospatial Function Across Cohorts eTable 10. Interactions by Sleep and APOE for Global Cognition eTable 11. Interactions by Sleep and Sex for Global Cognition eTable 12. Interaction by Sleep and Excessive Daytime Sleepiness and Global Cognition eReferences. [file jamanetwopen-e2325152-s001.pdf]

Supplemental Online Content

Pase MP, Harrison S, Misialek JR, et al. Sleep architecture, obstructive sleep apnea, and cognitive function in adults. *JAMA Netw Open*. 2023;6(7):e2325152. doi:10.1001/jamanetworkopen.2023.25152

eMethods. The Sleep and Dementia Consortium (SDC): Design and Overarching Aims

eTable 1. Analytic Sample Selection

eTable 2. Creation of Global Cognitive Composite Scores

eTable 3. Cognitive Domain Scores Used in Analyses

eTable 4. Means and SDs for Each Cognitive Test in Each Cohort Analysis Sample

eTable 5. Association Between Sleep and Attention and Processing Speed Across Cohorts

eTable 6. Association Between Sleep and Executive Function Across Cohorts

eTable 7. Association Between Sleep and Verbal Learning and Memory Across Cohorts

eTable 8. Association Between Sleep and Language Across Cohorts

eTable 9. Association Between Sleep and Visuospatial Function Across Cohorts

eTable 10. Interactions by Sleep and APOE for Global Cognition

eTable 11. Interactions by Sleep and Sex for Global Cognition

eTable 12. Interaction by Sleep and Excessive Daytime Sleepiness and Global Cognition

eReferences.

This supplemental material has been provided by the authors to give readers additional information about their work.

© 2023 Pase MP et al. *JAMA Network Open.*

eMethods: The Sleep and Dementia Consortium (SDC): Design and Aims

The SDC curates data from 5 community-based cohorts that have performed methodologically consistent, overnight, home-based polysomnography (PSG) and neurocognitive assessments. The cohorts include the Atherosclerosis Risk in Communities (ARIC) study, Cardiovascular Health Study (CHS), Framingham Heart Study (FHS), Osteoporotic Fractures in Men Study (MrOS), and Study of Osteoporotic Fractures (SOF). Our centralized PSG reading center has created harmonized sleep biomarkers across all studies and has made sleep data publicly accessible through the National Sleep Research Resource (NSRR). Moreover, PSG analytic tools have been made open-source via Luna. Combining data across studies with similar sleep exposure data and comprehensive cognitive assessments, the SDC maximizes statistical power and enables conclusions from study estimates across populations and settings. Through a series of sequential analyses, the SDC will investigate the temporal relationship between sleep, cognition, neurodegeneration and brain injury on MRI, and incident dementia by leveraging up to 21 years of dementia follow-up. The SDC will also examine how changes in sleep relate to dementia and related endophenotypes by examining changes in sleep across repeated PSGs available in four of our cohorts (ARIC, CHS, FHS, MrOS). In addition, all studies have extensive phenotyping, permitting a comprehensive examination of confounding and analysis of interactions (e.g., with the *APOE* genotype). Overall, the SDC aims to investigate sleep, cognition, and dementia risk comprehensively. Although this paper focuses on cognitive outcomes, other outcomes described in these eMethods will be examined in the future (e.g., risk of dementia).

Enrolment and cohort design methods

ARIC is a large, prospective study ongoing since 1987. ARIC was established to study cardiovascular disease in men and women from four geographically and racially diverse US communities: Minneapolis, MN; Washington County, MD; Forsyth County, NC; and Jackson, MS. A total of 15,792 participants (72% Whites) aged 45 to 64 years were enrolled at the baseline exam. PSGs were performed on a subset of participants from the Washington County, MD and suburban Minneapolis, MN sites. Participants are followed continuously for hospitalizations and death, and brief cognitive assessments were conducted at clinic visits 2 (1990-1992) and 4 (1996-1998). Between 2011 and 2013, all surviving ARIC participants were invited to

© 2023 Pase MP et al. *JAMA Network Open.*

complete a comprehensive dementia assessment (ARIC Neurocognitive Study) as part of the 5th clinic visit, and a subset underwent MRIs. Since then, dementia surveillance has included annual administration of the six-item screener and when appropriate, the Alzheimer's Disease 8 scale with informants. Additionally, comprehensive dementia ascertainment has been repeated at 2 in-person visits (visit 6: 2016-17 and visit 7: 2018-19) and by phone in 2020.

CHS is a prospective population-based cohort study of CVD in adults aged over 65 years. The four field centers are in Forsyth County, NC; Sacramento County, CA; Washington County, MD; and Pittsburgh, PA. The original cohort of 5,201 older adults was recruited from 1989 to 1990 from random samples of Medicare eligibility lists. An African-American cohort of 256 men and 431 women was added in 1992 from three of the four communities. Three of the four clinical centers participated in the SHHS (PA, CA, and MD). Persons were examined annually from enrolment to 1999 and continue to be monitored for morbidity and mortality. Annual exams have included a 30-minute screening cognitive battery. In 1992-94 and again in 1997-99, participants were invited to undergo brain MRI and detailed cognitive assessment (also performed annually thereafter) as part of the CHS Cognition Study.

FHS is an ongoing, longitudinal, community-based cohort study initiated in 1948 to investigate risk factors for CVD. The FHS now comprises three generations of participants: the Original cohort (Gen 1) followed since 1948, their Offspring (Gen 2) and spouses of these offspring, followed since 1971, and children from the largest Offspring families (Gen 3) enrolled in 2002.[1, 2] The Gen 2 cohort of 5,124 persons has been examined every four years. A multi-ethnic cohort of racially diverse adults was recruited in 1994-98 (Omni 1, N=507) and tested in parallel with the Gen 2 cohort. PSGs were performed on a subset of Gen 2 and Omni 1. From 1999, all surviving participants were invited to undergo brain MRI, with cognitive testing available from an earlier time point. Surveillance for dementia is ongoing, and decisions on dementia diagnosis and subtype are made at a consensus review that considers FHS neurologist exams, family interviews, and brain autopsy data.

MrOS recruited 5,995 community-dwelling men aged ≥65 years between 2000 and 2002 from 6 US sites (Birmingham, AL; Minneapolis, MN; Palo Alto, CA; Monongahela Valley near Pittsburgh, PA; Portland, OR; & San Diego, CA). From 2003 to 2005, participants were invited to the ancillary study titled Outcomes of Sleep Disorders in Older Men, consisting of a comprehensive sleep assessment (validated

© 2023 Pase MP et al. *JAMA Network Open.*

sleep questionnaire, in-clinic interview, and a series of clinical measurements, including home-based PSG). Of the original MrOS study, 56% of survivors were recruited into the ancillary sleep study reaching over 100% of the initial recruitment goal. In addition, participants were followed with multiple rounds of cognitive testing, and a follow-up PSG was performed from 2009-12.

SOF is a multisite cohort study of community-dwelling women.[3] The study recruited women aged 65 years or older who could walk unassisted. Participants were recruited from population-based listings in Baltimore County (MD), Minneapolis (MN), Portland (OR), and the Monongahela Valley (PA). In total, 9,704 women who were predominantly White were enrolled between 1986 and 1988, and 662 black women were enrolled between February 1997 and 1998. Between 2002 and 2004 (examination cycle 8), the SOF Sleep and Cognition ancillary study was established, incorporating 2732 participants from the study centers in Oregon and Pennsylvania.[4] A subsample of the SOF sleep and cognition sub-study was invited to undergo home-based PSG. In addition, participants were followed for cognitive impairment and completed neuropsychological assessments during follow-up study examination cycles.

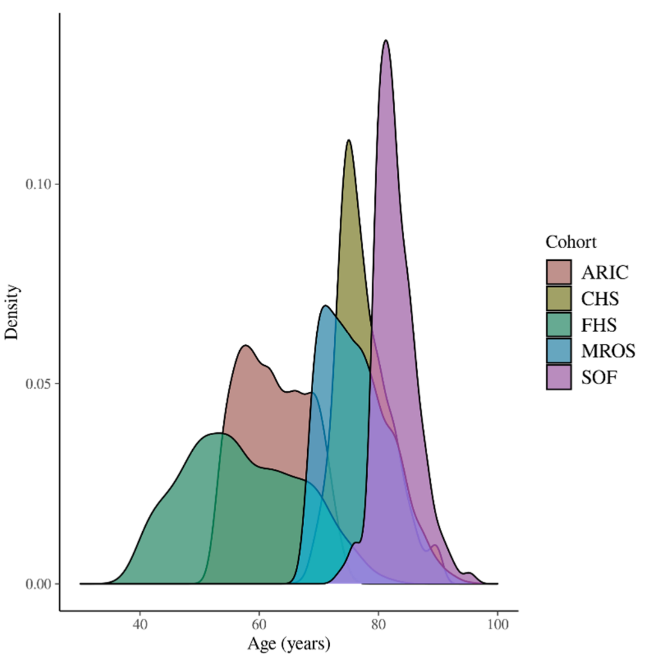

Age density plot by cohort for all participants with polysomnography. ARIC = Atherosclerosis Risk in Communities study; CHS = Cardiovascular Health Study; FHS = Framingham Heart Study; MrOS = Osteoporotic Fractures in Men Study; SOF = Study of Osteoporotic Fractures.

Assessment of brain MRI

Brain volumes on MRI are available for ARIC, CHS, and FHS. The acquisition and reading protocols have been described and harmonized previously as part of genetic consortia.[5, 6] All MRI scans were obtained using 1.5 or 3T field strength machines.

© 2023 Pase MP et al. *JAMA Network Open.*

The sequences include T1-weighted and either FLAIR or T2-weighted and T2 susceptibility-weighted imaging or T2-weighted gradient-recalled echo. Diffusion Tensor Imaging is also available within ARIC and the FHS, albeit over 10 years after the initial sleep study (a mean of 16 years for ARIC and 16.9 years for FHS).

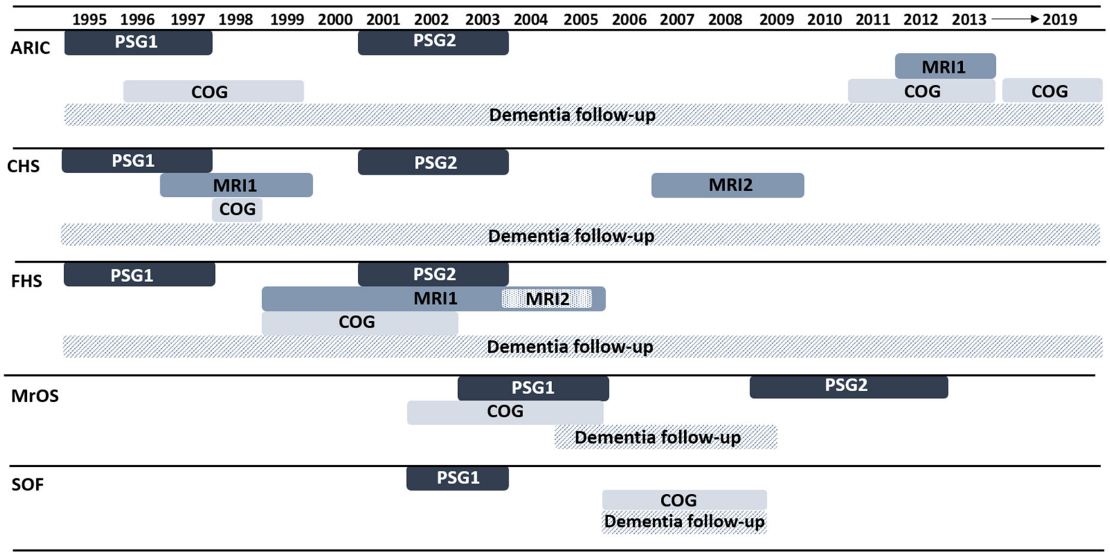

Overview of the sleep and brain health measures available across SDC cohorts. All cohorts except SOF underwent polysomnography (PSG1 and 2) at two time points (only the first PSG and cognitive data is used in the current paper). Magnetic resonance imaging (MRI1 and 2) assessments were performed at 1 time point in ARIC and two time points in CHS and FHS. All cohorts underwent cognitive (COG) assessments. Dementia follow-up was performed in each cohort with ARIC, CHS and FHS implementing continued and uninterrupted surveillance of incident dementia across their cohorts; MrOS and SOF recorded probable and prevalent dementia, respectively, at specific examination cycles. Several cohorts have performed cognitive testing and brain MRI after 2013.

ARIC = Atherosclerosis Risk in Communities study; CHS = Cardiovascular Health Study; FHS = Framingham Heart Study; MrOS = Osteoporotic Fractures in Men Study; SOF = Study of Osteoporotic Fractures; MRI1 = magnetic resonance imaging assessment 1;MRI2 = magnetic resonance imaging assessment 2; PSG1 = polysomnography study 1; PSG2 = polysomnography study 2; COG = neurocognitive assessments.

© 2023 Pase MP et al. *JAMA Network Open.*

Dementia case ascertainment

ARIC, CHS, FHS, and SOF have adjudicated dementia diagnosis by study-specific methods, based on varying combinations of neurocognitive data, informant interview, and hospitalization records, depending on the cohort, and based broadly on the Diagnostic and Statistical Manual of Mental Disorders, 4th edition (DSM-IV) criteria. In addition, MrOS investigators have adjudicated clinically significant cognitive impairment during follow-up by a report of physician-diagnosed dementia, use of dementia medication, or a change in modified Mini-Mental State Examination scores ≥ 1.5 standard deviations worse than the mean change from baseline to any follow-up visit. [7]

Race and ethnicity demographics

Query:

For the SOF cohort: Race (which included Hispanic) was asked at the baseline SOF visit (1986-1988), then at SOF visit 6 (1996-1998) a black cohort was added. The data used in this paper is from SOF visit 8 (2002-2004) and SOF visit 9 (2007-2008).
For MrOS cohort: Race/ethnicity was asked at the baseline visit (2000-2002). The data used in this study is from the sleep visit 1 (2003-2005)
For CHS cohort: There were separate questions—one for Hispanic origin (yes/no), and one for race (White, Black, American Indian/Alaskan native, Asian/Pacific Islander, other).

For the ARIC cohort: ARIC participants self-reported their race in mutually exclusive categories at baseline. Categories are here: Asian; Black; American Indian or Alaskan Indian: White.

For FHS, race was asked by self-report in the initial interview form: response options were white; black; Hispanic, Asian Indian or pacific islander; American Indian.

Source and categories:

SOF: self-report as follows. Q. What is your racial background? 1. Hispanic or Latino; 2. Asian or Pacific Islander; 3. African American; 4. White (Caucasian) 5. Another group not listed (Please say which)

© 2023 Pase MP et al. *JAMA Network Open.*

MrOS: Self-report as follows. Which of the following best describes your racial background (mark all that apply): White; Black or African American; Asian, Hispanic or Latino; American Indian or Alaskan Native; Native Hawaiian or Other Pacific Islander

CHS: The race/ethnicity data were obtained via interview as part of an eligibility form.

ARIC: ARIC participants self-reported their race in mutually exclusive categories at baseline. Categories are here: Asian; Black; American Indian or Alaskan Indian: White.

For FHS, race was asked by self-report in the initial interview form: response options were white; black; Hispanic, Asian Indian or pacific islander; American Indian.

<u>Categories not available:</u>

MroS: Any categories not populated were not specifically queried on the questionnaire.

SOF: Any categories not populated were not specifically queried on the questionnaire.

CHS: The race/ethnicity options are outlined in the response to #1 above

ARIC: Response options provided in Q1.

FHS: response options detailed above.

<u>Multiracial and multiethnic categories:</u>

In some cases, it was possible for participants to select multiple categories or to select multiracial as a response to ‘other.’ For ARIC, Multiracial or multicultural is not relevant, since categories were mutually exclusive.

© 2023 Pase MP et al. *JAMA Network Open.*

eTable 1. Analytic Sample Selection

	ARIC	CHS	FHS	MrOS	SOF
Participants with PSG and NP, n	1,879	836	756	2799	243
Exclusions, n					
<180 min of TST or <1 min of REM	48	43	13	77	4
Aged < 45 years	0	0	66	0	0
Prevalent dementia	3	2	6	0	0
Prevalent stroke	34	25	17	103	44
Missing covariates	3	65	14	0	0
Final sample, n	1,791	701[a]	640	2619	195

[a]Sample available for global cognition, a sub-sample of which had more extensive cognitive testing for the analysis of cognitive domains (n=232)

ARIC = Atherosclerosis Risk in Communities study; CHS = Cardiovascular Health Study; FHS = Framingham Heart Study; MrOS = Osteoporotic Fractures in Men Study; NP = neuropsychological testing; PSG = polysomnography; TST = total sleep time; REM = rapid eye movement; SOF = Study of Osteoporotic Fractures (SOF).

© 2023 Pase MP et al. *JAMA Network Open.*

eTable 2. Creation of the global cognitive composite scores

Cohort<br>Cognitive task	Transformation	Standardizing formula[a]	Component loading[b]
**ARIC**			
Delayed WR test (WR), N correct		(WR-6.59)/1.83	0.38
DSST, N correct		(DSST-43.61)/13.41	0.48
Verbal Fluency (VF), N correct		(VF-33.48)/12.49	0.45
**CHS**			
Modified MMSE (3MS), N correct		Z scored previously	0.69
DSST, N correct		Z scored previously	0.79
BVRT, N correct		Z scored previously	0.67
TMT-A, seconds	-log (TMT-A)	Z scored previously	0.67
TMT-B, seconds	-log (TMT-B)	Z scored previously	0.81
**FHS**			
TMT-B, seconds	-log (TMT-B)	(TMT-B+4.32)/0.45	0.35
Logical Memory (LM)[c], N correct		(LM-22.08)/6.75	0.31
Visual Reproductions (VR)[c], N correct		(VR-17.22)/6.33	0.37
Similarities (Sim), N correct		(Sim-16.75)/3.55	0.35
**MrOS**			
TMT-B, seconds	-log (TMT-B)	(TMT-B+6.27)/0.28	0.41
Digit Vigilance (DV), seconds	-log (DV)	(DV+4.71)/6.75	0.48
Modified MMSE (3MS), N correct	(3MS)**3	(3MS -815125)/129120	0.41
**SOF**			
Digit Span Forward (DSF), N correct		(DSF -7.44)/2.10	0.18
CVLT short form (CVLT), N correct		(CVLT -24.38)/4.78	0.45
TMT-B, seconds	-log (TMT-B)	(TMT-B+5.03)/0.47	0.40
Fluency vegetables (Veg), N correct		(Veg -7.44)/2.10	0.41

[a]Transformed cognitive tasks were used to create the standardized variables, where applicable.
[b]Global cognitive score calculated by summing the products of the standardizing formulas and the component loadings for each cognitive task
[c]Immediate and Delayed recall scores were summed together into a single variable
ARIC = Atherosclerosis Risk in Communities study; BVRT = Benton visual retention test; CHS = Cardiovascular Health Study; CVLT = California verbal learning test; DSST = Digit symbol substitution test; FHS = Framingham Heart Study; MrOS = Osteoporotic Fractures in Men Study; MMSE = mini-mental state examination; SOF = Study of Osteoporotic Fractures (SOF); TMT-A = Trail Making Test – part A; TMT-B = Trail Making Test – part B; WR = word recall

© 2023 Pase MP et al. *JAMA Network Open.*

eTable 3. Cognitive domain scores used in analyses

Cohort	Global Cognition	Attention & Processing Speed	Verbal Learning & Memory	Executive Function	Visuospatial Function	Language
**ARIC**	*	• DSST (N correct)	• Delayed word recall test (N correct)	• Verbal fluency test (F, A, S)		
**CHS**	*	• Digit Span (forward),<br>• TMT-A (completion time)	• CVLT long delay-free recall (N correct)	• Raven's Colored Progressive Matrices,<br>• Phonemic Fluency (sum of F and S, N correct),<br>• Digit Span backward (N correct),<br>• Stroop test (N correct),<br>• TMT-B (completion time)	• Rey-Osterreith Figure Immediate and Delayed Recall (N correct)<br>• Block design (N correct)	• Modified BNT (N, correct)<br>• Semantic Fluency (N, correct)
**FHS**	*	• TMT-A (completion time),<br>• Digit Span forward (N correct)	• PAL delayed recall (N correct)<br>• Logical Memory delayed recall (N correct)	• Similarities (N correct)<br>• TMT-B (completion time)<br>• Phonemic Fluency (F, A, S; N correct)<br>• Digit Span backward (N correct)	• HVOT (N correct)<br>• Visual Reproductions delayed recall (N correct)	• BNT (N correct)
**MrOS**	**	• Digit Vigilance (N correct)		• TMT-B (completion time)		
**SOF**	**	• Digit Span forwards (N correct)	• CVLT-short form	• Phonemic Fluency (F; N correct)<br>• TMT-B (completion time)<br>• Digit Span backward (N correct)		• Semantic Fluency (vegetables; N correct)

*ARIC, CHS, and FHS used Global Cognitive Scores derived from the CHARGE consortium[1].
**For MrOS and SOF, Global Cognition was calculated based on the first principal component from all tests. The 3MT also contributed to global cognition scores in MrOS.
Notes: All cognitive tests were performed within 5 years of PSG. Domain scores were created by calculating z-scores (based on cohort sample mean) and averaging scores within each domain. Appropriate transformations were applied, and speeded outcomes were multiplied by -1 such that higher scores indicate better performance on all tasks. ARIC = Atherosclerosis Risk in Communities study; BNT = Boston Naming Test; CHS = Cardiovascular Health Study; CVLT = California Verbal Learning Test; DSST = Digit Symbol Substitution Test; FHS = Framingham Heart Study; HVOT = Hooper Visual Organization Test; MrOS = Osteoporotic Fractures in Men Study; PAL = Paired Associate Learning; SOF = Study of Osteoporotic Fractures (SOF); TMT = Trail Making Test.

© 2023 Pase MP et al. *JAMA Network Open.*

eTable 4. Means and standard deviations for each cognitive test in each cohort's analysis sample (Vales are raw – untransformed and unstandardized)

	ARIC	CHS	FHS	MrOS	SOF
3MS (Total score)				94.0 [91.0, 97.0]	
Benton VRT (N correct)		4.47 (2.12)			
Block design (N correct),		10.44 (5.23)[a]			
(Modified) BNT (N correct)		25.61 (3.87)[a]			
BNT (N correct out of 30)			26.15 (4.18)		
CVLT long delay-free recall (N correct)		8.27 (3.20)[a]			
CVLT-Short Form (N correct)					24.6 (5.00)
Digit Span Forward (N correct)		7.93 (2.28)[a]	6.67 (1.50)		7.35 (1.95)
Digit Span Backward (N correct)		5.77 (2.23)[a]	4.58 (1.62)		
Digit Vigilance (Seconds)				505.0 [438.0, 599.0]	
DSST (N correct)	48.73 (10.69)	43.34 (12.50)			
Delayed word recall test (N correct)	6.80 (2.70)				
HVOT (N correct)			25.25 [23, 27][b]		
Logical Memory DR (N correct)			10.00 (3.49)		
PAL DR (N correct)					
Raven's Colored Progressive Matrices (N correct)		26.45 (5.72)[a]			
Rey-Osterreith Figure Immediate + DR (N correct)		27.89 (10.15)[a]			
Semantic Fluency (N, correct)		31.01 (9.80)[a]			
Similarities (N correct)			16.02 (3.89)		
Stroop test (N correct)		68.62 (24.26)[a]			
Trail Making Test B (Seconds)		144.46 (85.00) 118.12 (55.81)[a]	77.5 [61.0, 107.0][b]	107.0 [83.0, 138.0]	138.0 [98.0, 195.6]
Trail Making Test A (Seconds)		53.09 (28.69) 49.82 (24.31)[a]	33.0 [25.8, 40.8][b]		
Verbal Fluency (F; N correct)					11.1 (3.2)
Verbal Fluency (Sum of F, A, S, N correct)	36.39 (11.20)		30.17 (12.69)		
Verbal Fluency (sum of F and S, N correct)		24.46 (9.29)[a]			
Visual Reproductions DR (N correct)			7.83 (3.26)		

a CHS subsample with more extensive cognitive testing; b median (Q1, Q3).
3MS = modified mini mental state examination; ARIC = Atherosclerosis Risk in Communities study; BNT = Boston naming test; CHS = Cardiovascular Health Study; CVLT = California verbal learning test; DR = delayed recall; DSST = digit symbol substitution test; FHS = Framingham Heart Study; HVOT = Hooper visual organization test; MrOS = Osteoporotic Fractures in Men Study; PAL = paired associate learning; SOF = Study of Osteoporotic Fractures (SOF); VRT = visual retention test.
Note: For some CHS tests, two sets of value are provided because such tests were administered to participants at annual visit (for full sample, included in global cognition score) and at ancillary Cognition Study (for domain-specific analyses)

© 2023 Pase MP et al. *JAMA Network Open.*

eTable 5. Association Between Sleep and Attention and Processing Speed Across Cohorts

	ARIC (n=1,791)		CHS (n=227)		FHS (n=640)		MrOS (n=2619)		SOF (n=195)		Pooled Effect (n=5472)[c]	
	B (95%CI)	p	B (95%CI)	p	B (95%CI)	p	B (95%CI)	p	B (95%CI)	p	B (95%CI)	p
**Continuous predictors[a]**												
N1, % of TST	-0.07 (-0.52, 0.37)	0.75	-0.01 (-0.13, 0.10)	0.84	-0.42 (-1.44, 0.59)	0.41	-0.22 (-0.73, 0.29)	0.40	2.09 (-0.22, 4.41)	0.08	-0.04 (-0.21, 0.13)	0.64
N2, % of TST	-0.04 (-0.35, 0.26)	0.79	-0.002 (-0.01, 0.01)	0.68	-0.25 (-0.94, 0.44)	0.48	-0.06 (-0.45, 0.33)	0.75	1.53 (0.37, 2.69)	0.01	0.01 (-0.20, 0.21)	0.96
N3, % of TST	0.01 (-0.21, 0.24)	0.90	0.02 (-0.04, 0.08)	0.48	-0.14 (-0.68, 0.40)	0.61	-0.06 (-0.31, 0.20)	0.67	-1.38 (-2.37, -0.39)	0.007	-0.05 (-0.21, 0.12)	0.59
REM, % of TST	0.44 (-0.11, 0.98)	0.12	-0.001 (-0.01, 0.01)	0.92	1.26 (-0.01, 2.54)	0.05	0.84 (0.27, 1.42)	0.004	-1.32 (-3.29, 0.64)	0.19	0.38 (-0.16, 0.91)	0.17
SME, %	0.01 (-0.04, 0.07)	0.66	0.05 (-0.10, 0.19)	0.52	0.01 (-0.12, 0.14)	0.85	0.05 (-0.02, 0.13)	0.18	-0.05 (-0.31, 0.22)	0.74	0.03 (-0.02, 0.07)	0.22
WASO, min	0.003 (-0.04, 0.05)	0.90	-0.04 (-0.71, 0.62)	0.49	-0.01 (-0.12, 0.10)	0.86	-0.06 (-0.12, -0.002)	0.05	0.08 (-0.13, 0.30)	0.45	-0.02 (-0.05, 0.02)	0.33
**Categorical predictors[b]**												
Mild to Severe OSA vs. none	-0.06 (-0.13, 0.01)	0.12	0.06 (-0.01, 0.13)	0.58	-0.06 (-0.23, 0.10)	0.45	-0.05 (-0.12, 0.03)	0.25	0.23 (-0.05, 0.51)	0.12	-0.01 (-0.08, 0.06)	0.81
Moderate to Severe OSA vs. none	-0.01 (-0.10, 0.08)	0.83	0.29 (-0.04, 0.62)	0.01	0.05 (-0.16, 0.26)	0.64	-0.36 (-0.45, -0.28)	0.39	0.28 (-0.06, 0.63)	0.10	0.02 (-0.23, 0.26)	0.88
T90	-0.03 (-0.11, 0.04)	0.42	-0.06 (-0.25, 0.13)	0.53	-0.11 (-0.28, 0.06)	0.22	-0.003 (-0.007, 0.001)	0.13	-0.005 (-0.02, 0.01)	0.58	-0.004 (-0.01, 0.003)	0.28
TST, 6-9 hours vs. ≤6 (ref)	0.08 (0.02, 0.15)	0.01	0.08 (-0.10, 0.27)	0.38	0.09 (-0.06, 0.25)	0.23	0.04 (-0.03, 0.11)	0.29	0.05 (-0.22, 0.33)	0.70	0.07 (0.02, 0.11)	0.003

[a]For continuous measures, effects are interpreted per unit change in each sleep metric relative to a standard deviation unit change in the global composite score.
[b]For categorical measures, effects are interpreted as the difference in the global composite score (standard deviation units) between the specified group and the remainder of the sample.
[c]Combined effect obtained from random effects meta-analysis of all cohorts.
Notes: Higher scores indicate superior global cognitive performance. All results are adjusted for effects of age, age-squared, sex, education, the time interval between PSG and neuropsychological assessment, BMI, antidepressant use, and sedative use.
ARIC = Atherosclerosis Risk in Communities study; CHS = Cardiovascular Health Study; FHS = Framingham Heart Study; Mild + OSA = Apnea Hypopnea Index ≥ 5 vs referent (<5); Moderate + OSA = Apnea Hypopnea Index ≥ 15 vs referent (<15); MrOS = Osteoporotic Fractures in Men Study; REM = rapid eye movement sleep; SE = standard error; SME = sleep maintenance efficiency; SOF = Study of Osteoporotic Fractures (SOF); T90 = percentage of sleep time with oxygen saturation below 90% (<1% vs. ≥1 of total sleep time); TST = total sleep time; WASO = wake after sleep onset.

© 2023 Pase MP et al. *JAMA Network Open.*

eTable 6. Association Between Sleep and Executive Function Across Cohorts

Sleep predictors	ARIC (n=1,791)		CHS (n=195)		FHS (n=640)		MrOS (n=2619)		SOF (n=159)		Pooled Effect (n=6106)[c]	
	B (95%CI)	p	B (95%CI)	p	B (95%CI)	p	B (95%CI)	p	B (95%CI)	p	B (95%CI)	p
**Continuous predictors[a]**												
N1, % of TST	0.21 (-0.36, 0.79)	0.47	-0.02 (-0.13, 0.10)	0.78	-0.81 (-1.56, -0.07)	0.03	-0.51 (-1.00, -0.02)	0.04	1.03 (-1.49, 3.55)	0.42	-0.20 (-0.57, 0.17)	0.29
N2, % of TST	0.06 (-0.33, 0.46)	0.75	0.0005 (-0.01, 0.01)	0.89	-0.22 (-0.72, 0.28)	0.39	0.03 (-0.34, 0.40)	0.89	0.06 (-1.22, 1.33)	0.93	0.0005 (-0.009, 0.01)	0.92
N3, % of TST	-0.08 (-0.37, 0.21)	0.59	-0.01 (-0.07, 0.04)	0.65	0.13 (-0.26, 0.52)	0.52	-0.04 (-0.28, 0.20)	0.73	-0.32 (-1.42, 0.77)	0.56	-0.01 (-0.07, 0.04)	0.59
REM, % of TST	0.007 (-0.70, 0.71)	0.98	0.01 (-0.09, 0.10)	0.45	0.66 (-0.27, 1.60)	0.16	0.74 (0.19, 1.29)	0.008	0.93 (-1.21, 3.07)	0.4	0.30 (-0.11, 0.71)	0.15
SME, %	-0.03 (-0.11, 0.04)	0.38	0.10 (-0.06, 0.26)	0.19	0.06 (-0.03, 0.16)	0.19	0.10 (0.03, 0.18)	0.005	0.15 (-0.14, 0.44)	0.32	0.06 (-0.01, 0.12)	0.11
WASO, min	0.02 (-0.04, 0.08)	0.44	-0.08 (-0.20, 0.04)	0.21	-0.06 (-0.13, 0.02)	0.16	-0.10 (-0.15, -0.04)	0.001	0.03 (-0.20, 0.25)	0.82	-0.04 (-0.10, 0.01)	0.14
**Categorical predictors[b]**												
Mild to Severe OSA vs. none	0.002 (-0.09, 0.09)	0.97	-0.10 (-0.29, 0.09)	0.32	-0.04 (-0.16, 0.08)	0.53	-0.06 (-0.13, 0.02)	0.13	-0.007 (-0.31, 0.30)	0.96	-0.04 (-0.09, 0.01)	0.13
Moderate to Severe OSA vs. none	-0.03 (-0.14, 0.08)	0.61	0.06 (-0.01, 0.12)	0.63	-0.03 (-0.18, 0.12)	0.70	-0.04 (-0.12, 0.03)	0.27	-0.19 (-0.56, 0.18)	0.31	-0.006 (-0.06, 0.05)	0.84
T90	0.07 (-0.03, 0.16)	0.17	0.06 (-0.12, 0.23)	0.53	0.03 (-0.09, 0.16)	0.61	-0.002 (-0.006, 0.002)	0.38	0.004 (-0.01, 0.02)	0.67	-0.0005 (-0.007, 0.006)	0.89
TST, 6-9 hours vs. ≤6 (ref)	-0.03 (-0.11, 0.06)	0.56	0.09 (-0.09, 0.27)	0.51	-0.0003 (-0.11, 0.11)	1.0	0.03 (-0.04, 0.10)	0.44	-0.05 (-0.33, 0.24)	0.75	0.009 (-0.04, 0.06)	0.70

[a]For continuous measures, effects are interpreted per unit change in each sleep metric relative to a standard deviation unit change in the global composite score.
[b]For categorical measures, effects are interpreted as the difference in the global composite score (standard deviation units) between the specified group and the remainder of the sample.
[c]Combined effect obtained from random effects meta-analysis of all cohorts.
Notes: Higher scores indicate superior global cognitive performance. All results are adjusted for effects of age, age-squared, sex, education, the time interval between PSG and neuropsychological assessment, BMI, antidepressant use, and sedative use.
ARIC = Atherosclerosis Risk in Communities study; CHS = Cardiovascular Health Study; FHS = Framingham Heart Study; Mild + OSA = Apnea Hypopnea Index ≥ 5 vs referent (<5); Moderate + OSA = Apnea Hypopnea Index ≥ 15 vs referent (<15); MrOS = Osteoporotic Fractures in Men Study; REM = rapid eye movement sleep; SE = standard error; SME = sleep maintenance efficiency; SOF = Study of Osteoporotic Fractures (SOF); T90 = percentage of sleep time with oxygen saturation below 90% (<1% vs. ≥1 of total sleep time); TST = total sleep time; WASO = wake after sleep onset.

© 2023 Pase MP et al. *JAMA Network Open.*

eTable 7. Association Between Sleep and Verbal Learning and Memory Across Cohorts

	ARIC (n=1,791)		CHS (n=227)		FHS (n=640)		SOF (n=195)		Pooled Effect (n=2853)[c]	
	B (95%CI)	p	B (95%CI)	p	B (95%CI)	p	B (95%CI)	p	B (95%CI)	p
**Continuous predictors[a]**										
N1, % of TST	-0.21 (-1.17, 0.75)	0.67	-0.01 (-0.16, 0.15)	0.92	-0.33 (-1.16, 0.50)	0.43	-0.61 (-3.00, 1.78)	0.62	-0.03 (-0.18, 0.12)	0.73
N2, % of TST	-0.45 (-1.10, 0.20)	0.18	-0.01 (-0.02, 0.004)	0.25	-0.18 (-0.75, 0.38)	0.52	0.11 (-1.11, 1.33)	0.86	-0.007 (-0.02, 0.01)	0.30
N3, % of TST	0.26 (-0.22, 0.74)	0.28	0.03 (-0.08, 0.14)	0.44	0.19 (-0.25, 0.63)	0.39	-0.14 (-1.17, 0.90)	0.80	0.05 (-0.05, 0.15)	0.35
REM, % of TST	0.20 (-0.98, 1.38)	0.74	<0.001 (-0.02, 0.02)	1.00	0.10 (-0.94, 1.15)	0.85	0.41 (-1.64, 2.46)	0.69	0.0001 (-0.02, 0.02)	0.99
SME, %	0.06 (-0.06, 0.19)	0.30	-0.04 (-0.24, 0.16)	0.69	0.05 (-0.06, 0.15)	0.37	-0.05 (-0.32, 0.22)	0.72	0.04 (-0.04, 0.11)	0.33
WASO, min	-0.06 (-0.16, 0.04)	0.26	0.03 (-0.13, 0.20)	0.69	-0.06 (-0.14, 0.03)	0.20	-0.09 (-0.33, 0.15)	0.45	-0.05 (-0.11, 0.01)	0.12
**Categorical predictors[b]**										
Mild to Severe OSA, vs. none	-0.19 (-0.35, -0.03)	0.02	0.07 (-0.21, 0.34)	0.64	0.02 (-0.11, 0.16)	0.74	0.32 (0.03, 0.61)	0.03	0.03 (-0.16, 0.21)	0.76
Moderate to Severe OSA, vs. none	-0.08 (-0.28, 0.12)	0.41	-0.04 (-0.35, 0.27)	0.81	-0.02 (-0.19, 0.15)	0.79	0.09 (-0.26, 0.45)	0.61	-0.03 (-0.15, 0.08)	0.57
T90	-0.13 (-0.29, 0.03)	0.11	0.12 (-0.13, 0.38)	0.35	-0.03 (-0.17, 0.12)	0.71	0.005 (-0.01, 0.02)	0.99	-0.007 (-0.06, 0.05)	0.81
TST, 6-9 hours vs. ≤6 (ref)	-0.09 (-0.22, 0.05)	0.23	0.02 (-0.23, 0.27)	0.87	0.004 (-0.12, 0.13)	0.95	0.05 (-0.25, 0.35)	0.75	-0.02 (-0.11, 0.06)	0.58

[a]For continuous measures, effects are interpreted per unit change in each sleep metric relative to a standard deviation unit change in the global composite score.
[b]For categorical measures, effects are interpreted as the difference in the global composite score (standard deviation units) between the specified group and the remainder of the sample.
[c]Combined effect obtained from random effects meta-analysis of all cohorts.
Notes: Higher scores indicate superior global cognitive performance. All results are adjusted for effects of age, age-squared, sex, education, the time interval between PSG and neuropsychological assessment, BMI, antidepressant use, and sedative use.
ARIC = Atherosclerosis Risk in Communities study; CHS = Cardiovascular Health Study; FHS = Framingham Heart Study; Mild + OSA = Apnea Hypopnea Index ≥ 5 vs referent (<5); Moderate + OSA = Apnea Hypopnea Index ≥ 15 vs referent (<15); MrOS = Osteoporotic Fractures in Men Study; REM = rapid eye movement sleep; SE = standard error; SME = sleep maintenance efficiency; SOF = Study of Osteoporotic Fractures (SOF); T90 = percentage of sleep time with oxygen saturation below 90% (<1% vs. ≥1 of total sleep time); TST = total sleep time; WASO = wake after sleep onset.

© 2023 Pase MP et al. *JAMA Network Open.*

eTable 8. Association Between Sleep and Language Across Cohorts

	CHS (n=227)		FHS (n=640)		SOF (n=195)		Pooled Effect (n=1062)[c]	
	B (95%CI)	p	B (95%CI)	p	B (95%CI)	p	B (95%CI)	p
**Continuous predictors[a]**								
N1, % of TST	0.06 (-0.06, 0.19)	0.33	0.04 (-0.89, 0.98)	0.93	1.10 (-1.28, 3.49)	0.36	0.06 (-0.06, 0.19)	0.31
N2, % of TST	-0.004 (-0.01, 0.004)	0.34	0.18 (-0.45, 0.82)	0.57	0.55 (-0.65, 1.75)	0.37	-0.004 (-0.01, 0.006)	0.44
N3, % of TST	0.01 (-0.05, 0.08)	0.66	-0.15 (-0.64, 0.35)	0.56	-0.44 (-1.47, 0.60)	0.41	-0.13 (-0.50, 0.24)	0.49
REM, % of TST	< -0.001 (-0.02, 0.01)	0.94	-0.08 (-1.25, 1.10)	0.90	-0.52 (-2.54, 1.50)	0.62	-0.0007 (-0.02, 0.02)	0.94
SME, %	-0.01 (-0.17, 0.15)	0.88	0.02 (-0.10, 0.14)	0.75	-0.02 (-0.29, 0.26)	0.89	0.005 (-0.08, 0.09)	0.91
WASO, min	0.01 (-0.12, 0.14)	0.88	-0.03 (-0.13, 0.07)	0.51	0.01 (-0.21, 0.23)	0.93	-0.01 (-0.09, 0.06)	0.70
**Categorical predictors[b]**								
Mild to Severe OSA vs. none	-0.04 (-0.26, 0.18)	0.73	-0.02 (-0.17, 0.13)	0.80	-0.09 (-0.37, 0.20)	0.56	-0.04 (-0.15, 0.08)	0.55
Moderate to Severe OSA vs. none	0.15 (-0.11, 0.40)	0.25	-0.06 (-0.26, 0.14)	0.56	0.05 (-0.30, 0.40)	0.78	0.02 (-0.12, 0.17)	0.74
T90	0.22 (0.01, 0.42)	0.04	0.09 (-0.07, 0.25)	0.26	0.005 (-0.01, 0.02)	0.61	0.07 (-0.05, 0.19)	0.23
TST, 6-9 hours vs. ≤6 (ref)	0.02 (-0.18, 0.22)	0.87	-0.08 (-0.22, 0.06)	0.28	-0.06 (-0.35, 0.22)	0.66	-0.05 (-0.16, 0.06)	0.36

[a]For continuous measures, effects are interpreted per unit change in each sleep metric relative to a standard deviation unit change in the global composite score.
[b]For categorical measures, effects are interpreted as the difference in the global composite score (standard deviation units) between the specified group and the remainder of the sample.
[c]Combined effect obtained from random effects meta-analysis of all cohorts.
Notes: Higher scores indicate superior global cognitive performance. All results are adjusted for effects of age, age-squared, sex, education, the time interval between PSG and neuropsychological assessment, BMI, antidepressant use, and sedative use.
CHS = Cardiovascular Health Study; FHS = Framingham Heart Study; Mild + OSA = Apnea Hypopnea Index ≥ 5 vs referent (<5); Moderate + OSA = Apnea Hypopnea Index ≥ 15 vs referent (<15); REM = rapid eye movement sleep; SE = standard error; SME = sleep maintenance efficiency; SOF = Study of Osteoporotic Fractures (SOF); T90 = percentage of sleep time with oxygen saturation below 90% (<1% vs. ≥1 of total sleep time); TST = total sleep time; WASO = wake after sleep onset.

© 2023 Pase MP et al. *JAMA Network Open.*

eTable 9. Association Between Sleep and Visuospatial Function Across Cohorts

	CHS (n=220)		FHS (n=640)	
	B (95% CI)	p	B (95% CI)	p
**Continuous predictors[a]**				
N1, % of TST	-0.05 (-0.18, 0.09)	0.50	-0.27 (-1.10, 0.57)	0.53
N2, % of TST	-0.005 (-0.01, 0.004)	0.30	-0.55 (-1.12, 0.02)	0.06
N3, % of TST	0.05 (-0.02, 0.12)	0.17	0.39 (-0.05, 0.83)	0.08
REM, % of TST	0.004 (-0.01, 0.02)	0.66	0.67 (-0.38, 1.72)	0.21
SME, %	0.02 (-0.15, 0.20)	0.78	0.06 (-0.05, 0.16)	0.28
WASO, min	-0.003 (-0.15, 0.15)	0.97	-0.04 (-0.13, 0.04)	0.33
**Categorical predictors[b]**				
Mild to Severe OSA, vs. none	-0.06 (-0.30, 0.17)	0.60	0.04 (-0.10, 0.17)	0.60
Moderate to Severe OSA, vs. none	-0.09 (-0.36, 0.18)	0.52	0.02 (-0.15, 0.20)	0.79
T90	0.06 (-0.16, 0.28)	0.59	0.03 (-0.12, 0.17)	0.73
TST, 6-9 hours vs. ≤6 (ref)	0.09 (-0.12, 0.31)	0.39	0.06 (-0.07, 0.19)	0.36

[a]For continuous measures, effects are interpreted per unit change in each sleep metric relative to a standard deviation unit change in the global composite score.
[b]For categorical measures, effects are interpreted as the difference in the global composite score (standard deviation units) between the specified group and the remainder of the sample.
[c]Combined effect obtained from random effects meta-analysis of all cohorts.
Notes: Higher scores indicate superior global cognitive performance. All results are adjusted for effects of age, age-squared, sex, education, the time interval between PSG and neuropsychological assessment, BMI, antidepressant use, and sedative use.
CHS = Cardiovascular Health Study; FHS = Framingham Heart Study; Mild + OSA = Apnea Hypopnea Index ≥ 5 vs referent (<5); Moderate + OSA = Apnea Hypopnea Index ≥ 15 vs referent (<15); REM = rapid eye movement sleep; SE = standard error; SME = sleep maintenance efficiency; T90 = percentage of sleep time with oxygen saturation below 90% (<1% vs. ≥1 of total sleep time); TST = total sleep time; WASO = wake after sleep onset.

© 2023 Pase MP et al. *JAMA Network Open.*

eTable 10. Interactions by sleep and APOE genotype for global cognition

	Interaction p value			
Predictor Cohort	ARIC	CHS	FHS	MrOS
N1, %	0.679	0.810	0.594	0.206
N2, %	0.542	0.943	0.819	0.759
SWS, %	0.549	0.575	0.658	0.962
REM, %	0.758	0.982	0.627	0.169
SME, %	0.557	0.739	0.184	0.522
WASO, min	0.858	0.854	0.241	0.372
Mild + OSA	0.081	0.590	0.660	0.279
Moderate + OSA	0.559	0.651	0.371	0.398
T90	0.161	0.796	0.476	0.765

ARIC = Atherosclerosis Risk in Communities study; CHS = Cardiovascular Health Study; FHS = Framingham Heart Study; Mild + OSA = Apnea Hypopnea Index ≥ 5 vs referent (<5); Moderate + OSA = Apnea Hypopnea Index ≥ 15 vs referent (<15); MrOS = Osteoporotic Fractures in Men Study; REM = rapid eye movement sleep; SE = standard error; SME = sleep maintenance efficiency; T90 = percentage of sleep time with oxygen saturation below 90% (<1% vs. ≥1 of total sleep time); TST = total sleep time; WASO = wake after sleep onset.

© 2023 Pase MP et al. *JAMA Network Open.*

eTable 11. Interactions by sleep and sex for global cognition

	Interaction p value		
Predictor Cohort	**ARIC**	**CHS**	**FHS**
N1, %	0.189	0.090	0.589
N2, %	0.441	0.826	0.064
SWS, %	0.359	0.099	0.138
REM, %	0.870	**0.014**	0.122
SMW, %	0.315	0.508	0.202
WASO, min	0.272	0.709	0.339
Mild + OSA, (vs. none)	0.198	0.541	0.298
Moderate + OSA, vs. none	0.476	0.132	0.249
T90	0.670	0.613	0.992

ARIC = Atherosclerosis Risk in Communities study; CHS = Cardiovascular Health Study; FHS = Framingham Heart Study; Mild + OSA = Apnea Hypopnea Index ≥ 5 vs referent (<5); Moderate + OSA = Apnea Hypopnea Index ≥ 15 vs referent (<15); REM = rapid eye movement sleep; SE = standard error; SME = sleep maintenance efficiency; T90 = percentage of sleep time with oxygen saturation below 90% (<1% vs. ≥1 of total sleep time); TST = total sleep time; WASO = wake after sleep onset.

Description: There was a significant interaction between REM sleep % and sex in the CHS (p = 0.014). When stratifying by sex in the CHS, there was a statistically significant positive relationship between REM sleep % and global cognition in females (β±SE = 0.018±0.007, p = 0.011) and a non-significant negative relationship between REM sleep % and global cognition in males (β±SE = -0.009±0.008, p = 0.252).

© 2023 Pase MP et al. *JAMA Network Open.*

eTable 12. Interaction by sleep and excessive daytime sleepiness (ESS scores ≥11) and global cognition

	Interaction p value				
Predictor	**ARIC.**	**CHS**	**FHS**	**MrOS.**	**SOF**
N1, %	0.889	0.417	0.355	0.346	0.741
N2, %	0.386	0.962	0.545	0.893	**0.043**
SWS, %	0.686	0.832	0.889	0.948	0.495
REM, %	0.860	0.590	0.880	0.089	0.307
SME, %	0.107	0.134	0.667	0.377	0.154
WASO, min	0.076	0.187	0.478	0.701	0.218
Mild + OSA (vs. none)	0.983	0.062	0.203	0.926	0.804
Mod + OSA (vs. none)	0.240	0.224	**0.006**	0.150	0.586
T90	0.763	0.881	0.267	0.346	**0.025**

ARIC = Atherosclerosis Risk in Communities study; CHS = Cardiovascular Health Study; ESS = Epworth Sleepiness Scale Scores; FHS = Framingham Heart Study; Mild + OSA = Apnea Hypopnea Index ≥ 5 vs referent (<5); Moderate + OSA = Apnea Hypopnea Index ≥ 15 vs referent (<15); MrOS = Osteoporotic Fractures in Men Study; REM = rapid eye movement sleep; SE = standard error; SME = sleep maintenance efficiency; SOF = Study of Osteoporotic Fractures (SOF); T90 = percentage of sleep time with oxygen saturation below 90% (<1% vs. ≥1 of total sleep time); TST = total sleep time; WASO = wake after sleep onset.

Description:
Interaction between OSA and ESS scores in the FHS. There was a significant interaction between moderate to severe OSA (versus none) and excessive daytime sleepiness (ESS≥11) when predicting global cognition in the FHS. When stratifying by daytime sleepiness, prevalent moderate to severe OSA (vs. none) was associated with poorer the global cognition in persons with ESS scores ≥11 (β±SE= -0.536±0.215, p = 0.014). There was a no association between prevalent moderate to severe OSA and global cognition in persons with ESS scores <11 (β±SE = 0.160±0.108, p = 0.138).

Interaction between N2% and ESS scores in SOF. There was a significant interaction between N2 % and excessive daytime sleepiness (ESS≥11) when predicting global cognition in the SOF. When stratifying by daytime sleepiness, higher N2 % was associated with poorer the global cognition in persons with ESS scores ≥11 (β±SE = -10.77±4.76, p = 0.428), although the association did not reach statistical significance. In contrast, higher N2% was associated with better global cognition in persons with ESS scores <11 (β±SE = 0.805±0.620, p = 0.196).

Interaction between T90 and ESS scores in SOF. There was a significant interaction between T90 (having ≥1% sleep time with SaO2 <90% vs. <1% [ref]) and excessive daytime sleepiness (ESS≥11) when predicting global cognition in the SOF. When stratifying by daytime sleepiness, prevalent T90 was associated with better global cognition in persons with (β±SE = 0.361±0.189, p = 0.098) and without (β±SE = 0.006±0.009, p = 0.510) ESS scores ≥11.

© 2023 Pase MP et al. *JAMA Network Open.*

eReferences

1. Tsao CW, Vasan RS. Cohort profile: The framingham heart study (fhs): Overview of milestones in cardiovascular epidemiology. *Int J Epidemiol*. 2015;44:1800-1813
2. Tsao CW, Vasan RS. The framingham heart study: Past, present and future. *Int J Epidemiol*. 2015;44:1763-1766
3. Cummings SR, Nevitt MC, Browner WS, Stone K, Fox KM, Ensrud KE, et al. Risk factors for hip fracture in white women. Study of osteoporotic fractures research group. *New England Journal of Medicine*. 1995;332:767-773
4. Spira AP, Blackwell T, Stone KL, Redline S, Cauley JA, Ancoli-Israel S, et al. Sleep-disordered breathing and cognition in older women. *Journal of the American Geriatric Society*. 2008;56:45-50
5. Bis JC, DeCarli C, Smith AV, van der Lijn F, Crivello F, Fornage M, et al. Common variants at 12q14 and 12q24 are associated with hippocampal volume. *Nat Genet*. 2012;44:545-551
6. Hibar DP, Adams HHH, Jahanshad N, Chauhan G, Stein JL, Hofer E, et al. Novel genetic loci associated with hippocampal volume. *Nature communications*. 2017;8:13624-13624
7. Satizabal CL, Beiser AS, Chouraki V, Chêne G, Dufouil C, Seshadri S. Incidence of dementia over three decades in the framingham heart study. *New England Journal of Medicine*. 2016;374:523-532
8. Lopez OL, Kuller LH, Fitzpatrick A, Ives D, Becker JT, Beauchamp N. Evaluation of dementia in the cardiovascular health cognition study. *Neuroepidemiology*. 2003;22:1-12
9. Tranah GJ, Blackwell T, Stone KL, Ancoli-Israel S, Paudel ML, Ensrud KE, et al. Circadian activity rhythms and risk of incident dementia and mild cognitive impairment in older women. *Ann Neurol*. 2011;70:722-732
10. Knopman DS, Gottesman RF, Sharrett AR, Wruck LM, Windham BG, Coker L, et al. Mild cognitive impairment and dementia prevalence: The atherosclerosis risk in communities neurocognitive study (aric-ncs). *Alzheimers Dement (Amst)*. 2016;2:1-11
11. Leng Y, Redline S, Stone KL, Ancoli-Israel S, Yaffe K. Objective napping, cognitive decline, and risk of cognitive impairment in older men. *Alzheimers Dement*. 2019;15:1039-1047

© 2023 Pase MP et al. *JAMA Network Open.*
