# Supplementary material for: Sleep Architecture, Obstructive Sleep Apnea, and Cognitive Function in Adults
Source: JAMA Netw Open. 2023 Jul 18;6(7):e2325152. doi: 10.1001/jamanetworkopen.2023.25152 (PMC10354680; doi:10.1001/jamanetworkopen.2023.25152)
Supplement: Supplement 2. — Data Sharing Statement [file jamanetwopen-e2325152-s002.pdf]

Data Sharing Statement

Pase. Sleep Architecture, Obstructive Sleep Apnea, and Cognitive Function in Adults.
JAMA Netw Open. Published online July 18, 2023. doi:10.1001/
jamanetworkopen.2023.25152

Data
Data available: Yes
Data types: Deidentified participant data
How to access data: SLeep data can be requested from NSRR: https://sleepdata.org/
Cogntiive data can be requested from each participating cohort or from resources such as BioLINCC: https://biolincc.nhlbi.nih.gov/home/
When available: beginning date: 03-31-2024

Supporting Documents
Document types: None

Additional Information
Who can access the data: researchers whose proposed use of the data has been approved
Types of analyses: For any purpose, pending site approval
Mechanisms of data availability: After approval of a proposal, or with a signed data access agreement
